# Oral immunotherapy for milk allergy: a systematic review

**DOI:** 10.1186/1710-1492-8-S1-A14

**Published:** 2012-11-02

**Authors:** Joanne P Yeung, Lorie A Kloda, Jason R McDevitt, Moshe Ben-Shoshan, Reza Alizadehfar

**Affiliations:** 1 Pediatric Allergy and Immunology, McGill University, Montreal, Quebec, Canada, H3H 1P3; 2 Libraries, McGill University, Montreal, Quebec, Canada, H3G 1Y6; 3 Faculty of Medicine, McGill University, Montreal, Quebec, Canada, H3A 1W9

## Background

Milk oral immunotherapy (MOIT) may be an alternative to avoidance in treatment of patients with IgE-mediated cow milk allergy (IMCMA). We aim to determine the effect of MIOT through a systematic review.

## Methods

Randomized controlled trials on MOIT were identified from 13 databases, conference proceedings, theses and unpublished trials, as part of a review with the Cochrane Collaboration. A total 1945 records were identified and screened. Children and adults with IMCMA were included. Studies were selected and methodological quality assessed independently by two reviewers.

## Results

138 records were reviewed and 13 were included, representing 4 trials. A total of 170 patients were studied (88 MOIT, 82 control). Two studies used blinding and 2 used an avoidance diet control. Fifty-two (59%) patients of the MOIT group were able to tolerate a full serving of milk (about 200 mL) compared to 7 (9%) of the control group (RR 6.05, 95^%^ CI 3.2, 11.44). In addition, 26 (30%) in the MOIT group could ingest a partial serving of milk (10-184 mL) while none could in the control group (RR 11.55, 95% CI 2.85, 46.87). None of the studies assessed the patients following a period off immunotherapy. Adverse reactions were common (79 of 88 had at least one symptom), although most were local and mild. For every 7 patients receiving MOIT, 1 required intramuscular epinephrine. One patient required it on 2 occasions.

## Conclusion

MOIT can lead to desensitization in the majority of individuals with IMCMA although the development of long-term tolerance has not been established.

